# The National Insurance Bill

**Published:** 1911-08-05

**Authors:** Claude Tickell

**Affiliations:** Myddylton House, Saffron Walden.


### INSTITUTIONAL LETTER-BOX.

### THE NATIONAL INSURANCE BILL.

*To the Editor of* The Hospital.

Sir,—Would not the medical profession be well advised to insist upon the conversion of the "medical and sanatorium benefits" into additional weekly payments, seeing that otherwise the fees they are to receive and the treatment they are to prescribe will be dictated to them by a lay officialdom dominated by a particular school of medicine and a desire to economise politically?—Yours truly,

Myddylton House, Claude Tickell.

Saffron Walden.

Secretary, National Anti-Vaccination League: We regret that we cannot publish the article forwarded, since it contains no new criticism.

